# Self-Supporting Dispensaries

**Published:** 1834-07-01

**Authors:** 


### SELF-SUPPORTING DISPENSARIES.

In our second Number we made a few observations on the advantages to be derived from these useful institutions, and we return to the subject with the greater pleasure, that some attempts (feeble and imperfect ones, but still attempts,) have been made to supply a deplorable void in the London practice of physic. The first that we heard of is called the Medico-Chirurgical Dispensary, situated in Crown-street, Soho; and one of its admission letters, now before us, informs us, that "this institution was established for the purpose of remedying the objections which at present apply to dispensaries, namely, the difficulty and loss of time incurred in procuring letters of admission. It is not supported by charitable contributions, but by a small charge to the patient of fourpence per day, or two shillings per week, which it is computed will repay the expenses attending it. When patients require to be seen at home, the charge will be double." We really see no objection to this method of paying a groat a day, by which a man in middling circumstances may enjoy the luxury of constant medical attendance for six pounds one shilling and eightpence per annum; but we are rather annoyed by a short sentence which follows: "A physician and surgeon attend daily at twelve o'clock." Now, although every medico-chirurgical dispensary cannot expect to be attended by physicians or surgeons of name in one sense, they ought to be of name in the other: for our own part, we should not like to be physicked or incised by men of no name at all. In truth, we would rather have pseudonymous than anonymous doctors; and we observe in authentic records, that, when Apollo was wont to wander upon earth, he always took up some name, and never practised under the title of ὁ δεινος, or Dr. ——. However, as six months have elapsed since we were favoured with the dispensary letter in question, we trust that this deficiency has been supplied.

We have likewise received the prospectus of another institution, accompanied by the following note: "*The surgeons of the St. Pancras Medical Association present their compliments to the Editor of the Medical Quarterly Review, and request to be favoured with his opinions in the next Number of that Journal.*"— We trust that we shall not be thought wanting in courtesy, far less in justice, if we state two most obvious objections to the plan of this institution. The first may be considered as a matter of form or ceremony, but it is not so. The only persons whose names appear on the face of the prospectus, as members of the association, are the eight medical men who intend to treat the patients. Now, it appears to us absolutely requisite that every scheme of this kind should be backed by some persons of high station; some persons, in fact, whose rank, though it by no means removes them from strife or dispute, at least elevates them far above the sphere of professional squabbles, and who are valuable to charitable societies, not only as patrons, but as umpires. Such persons may be compared to the weight in a clock, which, though apparently inert, is essential to the existence of a well-regulated movement. It is very possible that this objection, like the one we made to the Crown-street document, may have been annulled by the progress of the institution, but our second one is more important.

The medical men attached to this institution appear to be all of them general practitioners, and we find them telling us, in consequence, that "the surgeons constituting this association will dispense their own prescriptions, thus saving the cost of keeping up a dispensary house and appendages, and salary of dispensing apothecary; a charge equal to at least a third of the whole income of such institutions: in other words, of every guinea subscribed by the humane and excellent supporters of these charities, less than 14*s.* are appropriated to the literal benefit of the poor."—Surely this is very poor reasoning; for, if the tripartite division of the profession into physicians, surgeons, and druggists, be beneficial, (and few are crazed enough to deny it,) then, as Horace says, *Æque pauperibus prodest, locupletibus æque;* and the niggard economy which would deny the poor the benefit of the division of scientific labour is worthy of anything but applause. What should we say to a *gastronome* who should discharge his kitchen and scullery maid, under the shallow pretence that their wages might be "appropriated to the literal benefit" of his palate? We should say he was stark mad, and deserved never more to taste a savoury ragout. In small towns, as in small kitchens, a very minute division of labour cannot take place; but why should we be driven to shifts in London, and why, above all, in a dispensary, whose practice, conducted under the public eye, should be a model of all that is rational and scientific?

We could wish to see some societies of this kind intended for persons, who, though of a higher grade than those marked out by the St. Pancras Association, are yet wholly incapable of facing a doctor's bill. To a single man, living on 100*l.* a year, or a married man with an income of 250*l.*, must not the anticipation of a long doctor's bill be more bitter than all the columba and quassia on which it is founded? To such a man the existence of a society which, for a guinea a year paid for each member of his family, would ensure him against one (not the least) of his anxieties, would be a real blessing: it would be like insurances against fire, and, like them, would mark an era in civilization.

